# A Modified Delphi Study to Establish Essential Clinical Pharmacology Competencies

**DOI:** 10.1007/s43441-023-00609-y

**Published:** 2024-02-06

**Authors:** Bernadette Johnson-Williams, Kellie Reynolds, Joga Gobburu, Albert Rundio

**Affiliations:** 1 Office of Clinical Pharmacology, Office of Translational Sciences, Center for Drug Evaluation and Research, Food and Drug Administration, Silver Spring, MD USA; 2 School of Pharmacy, University of Maryland, Baltimore, 620 W. Lexington Street, Baltimore, MD 21201 USA; 3 College of Nursing and Health Professions, Drexel University, 60 N. 36th Street, Philadelphia, PA 19104, USA; 4 Center for Drug Evaluation and Research, 10903 New Hampshire Avenue, Silver Spring, MD 20993 USA

**Keywords:** Delphi study, Competencies, Clinical pharmacology, Drug development, Workforce development, Regulatory science

## Abstract

**Introduction:**

Competency-based education has been commonly used to enhance the healthcare workforce for some time. A translational discipline that is integral to drug development and impactful on healthcare and public health is clinical pharmacology. With such contribution, it is essential that the clinical pharmacology workforce is adequately equipped to address the demands of emerging trends of drug development.

**Objectives:**

The primary objective of this study was to determine the most significant competencies needed for a clinical pharmacologist in the regulatory environment.

**Methods:**

A two round modified Delphi technique was administered to 29 clinical pharmacologists within the Office of Clinical Pharmacology (OCP) between November 2021–January 2022.

A questionnaire consisting of core and technical competencies was administered electronically using SurveyMonkey ® to gain consensus about essential clinical pharmacology competencies. Participants used a Likert scale to rank importance of competencies from strongly agree (1), agree (2), neutral (3), disagree (4), strongly disagree (5). Participants also suggested topics to be included in the next round. Consensus was set at 60%. The competencies receiving the most consensus at 60% in round one and the new topics proceeded to the second round. In the second and final round, participants ranked the suggested competencies. Descriptive statistics and a McNemar change test were utilized to analyze data. Only data from the participants who completed both rounds was used in the study.

**Results:**

In round one participants ranked all fifty-six core and technical competencies as essential with consensus of at least 60%. In round two, participants ranked sixty-two competencies as essential with consensus of at least 60%. A McNemar change test demonstrated stability of ranking between rounds.

**Conclusion:**

Essential core and technical competencies can build education programs to sustain the emerging clinical pharmacology workforce in the Office of Clinical Pharmacology. The Delphi technique is a suitable approach to determine essential competencies because it cultivates consensus and gains insight from experts in the forefront of drug development.

**Supplementary Information:**

The online version contains supplementary material available at 10.1007/s43441-023-00609-y.

## Introduction

According to the Georgetown University Health Policy Institute [1] 66 percent of adults in the United States take prescription drugs. It is vital that our nation’s drug supply is both safe and effective. An area of healthcare that is cutting edge, translational among disciplines and integral to drug development is clinical pharmacology. With such an impact on public health, it is vital that the clinical pharmacology workforce is properly skilled and trained. A deficit in proper training and skills set will have a negative effect on the workforce’s ability to cope with the dynamic changes impacting drug development [2]. 

Many healthcare professionals support the use of competencies for education development. Competency is an ability that is observable and integrates several parts such as attitudes, knowledge, skill, and values according to Silva et al. [3] Competencies can be used to validate skills sets in workplace education and have been used in healthcare for quite some time. Competency-based education can significantly prepare the pharmaceutical physician and drug development scientific workforce for opportunities and challenges in the next decade [3]. Recognizing the importance of competency development, the Office of Personnel Management (OPM) launched a Federal Workforce Competency Initiative in April 2021 to ensure the federal workforce has the necessary skills to be successful by recruiting, training, and retaining its people [4]. The initiative will be administered in a series of phases focusing on core and technical competencies. As cutting-edge science emerges, competencies can enable professionals to remain current and well-equipped in the field.

Competencies in clinical pharmacology can develop current staff and the future pipeline [5]. Additionally, the development of a task force to explore the need for leadership competencies in clinical pharmacology can advance the workforce because of their interrelatedness among clinical discipline, science, policy, and public health [5]. The aim of this study was to determine the most significant competencies needed for a clinical pharmacologist in the regulatory environment. Our question sought to determine which competencies would receive the greatest amount of consensus among clinical pharmacologists to address the demands of drug development and emerging trends.

## Methods

### Delphi Technique

**Fig. 1:**
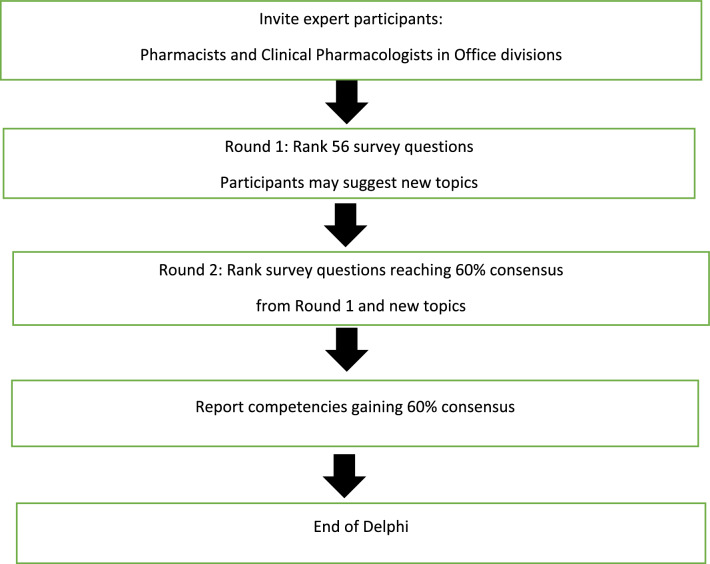
Diagram of the Delphi Technique. This figure depicts the iterative process in this Modified Delphi Study [6].

The study design used a modified Delphi technique as shown in Fig. 1.

This is because instead of initially generating questions in the first round, the study created questions from an existing knowledge matrix previously used in OCP based on Nieforth [7]. The Delphi technique permits effective decision making in healthcare and facilitates consensus in multiple stages [8]. It considers structured group communication where issues that are unknown or incomplete are evaluated by using an iterative process [9]. The objectives for Delphi techniques in health sciences are: “identifying the current state of knowledge; improving predictions of possible future circumstances; resolving controversial judgements; identifying and formulating standards or guidelines for theoretical and methodological issues; developing measurement tools and identifying indicators; formulating recommendations for action and prioritizing measures” [9] (p. 2). The Delphi technique is appropriate to obtain consensus by using questionnaires for data collection from a panel of subject matter experts [10].

### Study Location and Administration

The study location was in the Office of Clinical Pharmacology (OCP) at the Food and Drug Administration, Silver Spring, MD. The study was approved and determined as exempt research by the FDA and Drexel University Institutional Review Board because it was intended to enhance quality. The researcher discussed the purpose, goals, and contribution of the study with office leadership and subject matter experts by thoroughly explaining the study process and details. The researcher also emphasized the importance of the study to gain support and boost participation. To develop and review the survey questions, the researcher collaborated with subject matter experts for accuracy to gain intended responses and to ensure content validity. Additionally, the researcher prepared the Delphi study participants by explaining the purpose of the study, the amount of time the study required, the number of study rounds, and the outcome of information gained from the study [8]. 

### Participants

The study enrolled 34 participants, which is within the recommended number for panelists for a Delphi study [11]. The sampling approach used was purposive, a type of non-probability sampling [12]. The participants who served as the Delphi expert panel were clinical pharmacologists and reviewers within the divisions in OCP. As members of OCP, the participants have a vested interest and knowledge which can help increase content validity [8]. Staff in the Immediate Office, fellows, summer students, contractors, interns, and division level leadership who served as experts to develop and review study questions were excluded. All participants received management approval and completed informed consent forms. The participants who were selected because of their education, training, and experience in the discipline could serve as experts to appropriately address the research question. Additionally, participation from all of the divisions promoted diversity in subject matter expertise which is significant for robustness of findings and influential for quality of data [8]. 

### Questionnaire

The modified Delphi study consisted of a questionnaire which was administered electronically using SurveyMonkey ® in two rounds. The questionnaire began with a demographic section about participants’ degree, years of experience, length of time in OCP, length of time at the employer, and length of time in the field. Next, questions were categorized as core competencies and technical competencies with subdomains. The core competency domains were the following: communication, time management, negotiation skills, problem solving, customer service, and functional skills. The technical competency domains were the following: Drug Disposition, Pharmacology and Biomarkers, Quantitative Methods, Drug Safety, Pharmacotherapy, Clinical Trial Methods, and Guidance and Policy. Participants used a Likert scale to rank the importance of the competency from strongly agree (1), agree (2), neutral (3), disagree (4), strongly disagree (5). In the first round of questions, participants ranked the suggested competencies and suggested topics to be included in the next round [13]. The competencies receiving the most consensus at 60% and the new topics proceeded to the next round. In the second and final round, participants ranked the suggested competencies and competencies receiving the most consensus at 60% were determined the essential clinical pharmacology competencies. Only the information from the participants who completed all of the rounds was used in the study.

### Data Analysis

Descriptive and nonparametric statistics were used for data analysis. von der Gracht summarized consensus measurement by descriptive data and indicated consensus is meaningful if nominal or Likert scales are used to show agreement [14]. The level of agreement can be used as those suitable in political voting systems such as simple majority, two-third majority and absolute [14] and in this study, 60% indicated the majority. Nonparametric statistics were used because the questionnaire uses a Likert scale, and the measurement should analyze central tendency and level of dispersion [8]. Often Delphi studies use descriptive statistics with a central tendency. Analyzing the data over iterations allows the strength of consensus and merging of opinions [14]. Statistical Package for the Social Sciences (SPSS) (IBM® SPSS® Statistics Subscription) was used to analyze the data of the survey rounds.

A McNemar change test was conducted to determine the stability between the rounds. This was done between the same competencies that were ranked both in rounds one and two. The McNemar change test is a repeated measures test used with nominal data. The McNemar change test is appropriate when comparing the results of the same samples twice in Delphi studies [14]. In this study the consensus of the ranked competencies was compared between the rounds using the same participants. All competencies that ranked strongly agree or agree were coded to yes for consensus and all competencies ranked neutral, disagree, and strongly disagree were coded to no for non-consensus.

### Consensus

There is no standardization for consensus in the Delphi method and consensus spans from 51–100% [15]. According to Belton et al. the selection for consensus should provide confidence in the outcome suited to meet the needs of the research topic and has flexibility [11]. The researcher used 60% for consensus with the option to further analyze data for trends among the rounds. According to Pew research 60% is considered a large majority and considers any possible margin of error [16]. Since there is no standardization for consensus, the researcher determined consensus should be representative of the majority with allowance for further evaluation for trends in subsequent rounds. Furthermore, consensus agreement may not have to be set as high if there is not a critical decision and general opinion of trends may be more appropriate [11]. 

## Results

### Round One

#### Participant Demographics

Survey one was sent to thirty-four participants. Thirty-two of thirty-four participants completed round one of the Delphi study with a response rate of 94%. 8 (25%) respondents held a PharmD degree. 16 (50%) respondents held a PhD in pharmaceutical sciences. 8 (25%) respondents held a PhD in another discipline, e.g., chemical engineering, pharmacology and toxicology, biochemistry, pathobiology and molecular medicine, cytogenetics. One respondent with a PharmD also indicated an additional degree, a PhD in pharmaceutical sciences. 4 (12.5%) indicated having 0–3 years of experience in drug development. 9 (28.1%) indicated having 4–10 years of experience in drug development. 7 (22%) indicated having 11–14 years of experience in drug development. 12 (37.5%) indicated having more than 14 years of experience in drug development. 14 (43.8%) indicated having 0–5 years of experience at the Agency. 6 (18.8%) indicated having 6–10 years of experience, 11–14 years of experience and more than 14 years at the Agency, respectively. 15 (46.9%) respondents indicated having 0–5 years of experience in the Office of Clinical Pharmacology (OCP). 8 (25%) indicated having 6–10 years of experience in OCP. 4 (12.5%) indicated having 11–14 years of experience in OCP and 5 (15.6%) indicated having more than 14 years of experience in OCP. As shown in Table 1, responses were representative of the divisions in OCP.

**Table 1:** Review Divisions in the Office of Clinical Pharmacology

Divisions	% Responses
Divisions of Cancer Pharmacology (DCP I & II)	15.6% (5)
Division of Cardiometabolic and Endocrine Pharmacology (DCEP)	9.4% (3)
Division of Infectious Disease Pharmacology (DIDP)	9.4% (3)
Division of Immune and Inflammation Pharmacology (DIIP)	15.6% (5)
Division of Neuropsychiatric Pharmacology (DNP)	21.9% (7)
Division of Pharmacometrics (DPM)	9.4% (3)
Division of Translational and Precision Medicine (DTPM)	12.5% (4)
Division of Applied and Regulatory Science (DARS)	6.3% (2)

*N* = 32. The percentage may not equal 100% due to rounding

#### Competencies

All fifty-six competencies were rated as essential; 60% of participants or more rated strongly agree or agree (see Online Resource 1). There was a total of twenty-three core competencies and thirty-three technical competencies. The participants suggested six additional competencies: Domain: Negotiation: Uses listening skills when negotiating; Domain: Drug Disposition: Recognize that drug-drug interactions being translated through the understanding of the drug development program in certain therapeutic areas is essential to public health mission; Domain: Pharmacology and Biomarker: Identifies when to consider targeted biomarker variability, pediatric development variability or diversity of (ethnic/racial) population frequent of biomarker during negotiation of final communication in labeling with stakeholders; Domain: Quantitative Methods: Applies knowledge of disease frequency and prevalence to understand the full spectrum of safety/efficacy evaluation for decision making and communication; Domain: Drug Safety: Utilizes the most appropriate metrics and outcomes in analyses by working closely with drug safety and clinical teams and Apply relevant drug safety competencies in labeling.

### Round Two

#### Participant Demographics

Survey two was sent to thirty-two participants. Twenty-nine participants completed questions in round two with a response rate of 90.6%. 8 (27.6%) respondents held a PharmD. 13 (44.8%) respondents held PhD in Pharmaceutical Sciences. 8 (27.6%) respondents held a PhD in another discipline, e.g., Pharmacology, Chemical Engineering, Pathobiology and Molecular Medicine, Chemistry, Cytogenetics, Cell Biology and Biochemistry. 4 (13.8%) indicated having 0–3 years of experience in drug development. 9 (31.0%) indicated having 4–10 years of experience in drug development. 5 (17.2%) indicated having 11–14 years of experience in drug development. 11 (37.9%) indicated having more than 14 years of experience in drug development. 12 (41.4%) indicated having 0–5 years of experience at the Agency. 5 (17.2) indicated having 6–10 years of experience. 6 (20.7%) indicated having 11–14 years of experience and more than 14 years at the Agency, respectively. 13 (44.8%) respondents had 0–5 years of experience in the Office of Clinical Pharmacology (OCP). 5 (17.2%) indicated having 6–10 years of experience in OCP. 6 (20.70%) indicated having 11–14 years of experience in OCP and 5 (17.2%) indicated having greater than 14 years of experience in OCP. As seen in Table 2, responses were representative of the divisions in OCP.

**Table 2:** Review Divisions in the Office of Clinical Pharmacology

Divisions	% Responses
Divisions of Cancer Pharmacology (DCP I & II)	17.2% (5)
Division of Cardiometabolic and Endocrine Pharmacology (DCEP)	10.3% (3)
Division of Infectious Disease Pharmacology (DIDP)	6.90% (2)
Division of Immune and Inflammation Pharmacology (DIIP)	17.2% (5)
Division of Neuropsychiatric Pharmacology (DNP)	20.7% (6)
Division of Pharmacometrics (DPM)	10.3% (3)
Division of Translational and Precision Medicine (DTPM)	10.3% (3)
Division of Applied and Regulatory Science (DARS)	6.90% (2)

*N* = 29. The percentage may not equal 100% due to rounding

### Competencies

All sixty-two competencies which included the additional six competencies gained from round one were rated as essential; 60% of participants or more rated strongly agree or agree (see Online Resource 2). The following six additional competencies received consensus of 75% or greater: Question 15, Domain: Negotiation: Uses listening skills when negotiating received 96.4% consensus; Question 35, Domain: Drug Disposition: Recognize that drug-drug interactions being translated through the understanding of the drug development program in certain therapeutic areas is essential to public health mission received 93.1% consensus; Question 40, Domain: Pharmacology and Biomarker: Identifies when to consider targeted biomarker variability, pediatric development variability or diversity of (ethnic/racial) population frequent of biomarker during negotiation of final communication in labeling with stakeholders received 79.3% consensus; Question 46, Domain: Quantitative Methods: Applies knowledge of disease frequency and prevalence to understand the full spectrum of safety/efficacy evaluation for decision making and communication received 79.3%; Questions 52–53, Domain: Drug Safety: Utilizes the most appropriate metrics and outcomes in analyses by working closely with drug safety and clinical teams received 89% consensus and Apply relevant drug safety competencies in labeling received 75.9%.

#### Stability of Rounds One and Two

A McNemar change test was conducted to show stability between rounds [14]. A total of 29 participants ranked competencies for round one and round two. There were some instances where participants skipped responses. The competencies for both rounds one and two received consensus with at least 60% or higher. The results of the McNemar change test were not significant for all competencies, *p* > 0.05. This signifies stability of ranking between the rounds because there was no difference in consensus between rounds.

## Discussion

A well-designed Delphi study determining essential competencies can contribute to the field because it consults regulatory clinical pharmacologists who are at the intersection of drug development [14]. In this Delphi study participants ranked all competencies in rounds one and two as essential competencies in clinical pharmacology for success in the regulatory environment. Delphi studies are commonly done in two to three rounds [8, 9] and this study was concluded in two rounds instead of three because consensus was reached at 60% in the first round. This was attributed to several factors. The preliminary work done to administer the study survey contributed to study success. The researcher was not a clinical pharmacologist by training and remained neutral to administer the study [8]. The researcher modified statements from an existing knowledge matrix to develop the competencies for the study which was time efficient. Additionally, there was a working group consisting of the researcher and subject matter clinical pharmacologists from the office who drafted the questions for the competency study. An additional group of managers reviewed the questions developed by the working group for content and comprehensibility. This approach was similar to Schmalz et al [17] who indicated a well-planned Delphi study in can be done in two rounds.

Participants in this study ranked all competencies as essential with consensus of 60% or greater of strongly agree or agree as 60% is considered a large majority [16]. Most of the competencies in this study received consensus of 70% which is commonly used for consensus [9] or greater with a few exceptions (see online Resource 1 & 2). As 60% was the marker for consensus in this study and most competencies in the study received consensus of at least 70%, the competencies reaching consensus of 80% or greater as shown in Table 3 are considered to be the most significant [13].

**Table 3:** Competencies Receiving 80% Consensus in Rounds 1 and 2

Core competencies
Domain: Communication
1. Demonstrates effective writing and oral presentation/communication skills
2. Appropriately adjusts communication material to optimize understanding and utility by specific audiences (e.g., Industry, healthcare practitioner, public)
Domain: Time Management
3. Uses time effectively and efficiently
4. Prioritizes a wide range of tasks and projects in a timely manner
5. Demonstrates the ability to plan the workload for successful completion of projects
Domain: Negotiation Skills
6. Demonstrates the ability to negotiate skillfully in tough situations with both internal and external groups
7. Seeks win–win solutions to settle differences
8. Demonstrates skill in being direct and forceful as well as diplomatic when required
9. Builds trust between parties in negotiation
Domain: Problem Solving
10. Applies critical thinking to solve difficult problems with effective solutions
11. Researches broadly to solve a problem and with an open mind
12. Demonstrates sensitivity to potential unseen risks/threats during problem solving
13. Provides an objective analysis
Domain: Customer Service (Focus)
14. Strives to meet the needs, expectations, and requirements of internal coworkers and external stakeholders (e.g., industry, healthcare providers, press, congress, public) in a positive manner
15. Establishes and maintains effective relationships with coworkers and stakeholders and gains their trust and respect
16. Applies knowledge of direct internal and external feedback for improvement in the drug development and regulatory review process, policy development, and this information is communicated
17. Demonstrates dedication to stakeholder satisfaction
Domain: Functional/Skills
18. Performs in depth Clinical Pharmacology and sub-specialty reviews, e.g., quantitative pharmacology (pharmacokinetics and genomics) submissions in IND, NDA, BLAs, Supplements, and Amendments
19. Provides independent recommendations to review team regarding information related to the approvability of a clinical pharmacology package
20. Remains abreast/current in Clinical Pharmacology and new innovations in Clinical Pharmacology related to drug development
21. Maintains basic knowledge of other disciplines as appropriate
22. Applies up-to-date knowledge of Federal laws, FDA regulations, and related guidelines for industry applicable to the review process
23.Maintains relevant knowledge of CDER guidances, MaPPs and review division processes and procedures and OCP best practice documents
Technical Competencies
Domain: Drug Disposition
24. Assesses the clinical relevance of drug substance and formulation attributes to guide approvability, dosing/administration
25. Demonstrates knowledge of enzyme(s)/transporter(s) involved in drug disposition, including sources of variability
26. Translates knowledge of vitro, in vivo, and in silico methods to predict human metabolism to guide decision-making (labeling, post-marketing requests, etc.)
27. Evaluates and interprets effects of intrinsic and extrinsic factors on clinical PK
Domain: Pharmacology and Biomarkers
28. Demonstrates knowledge of various measures (and methods) for assessment of drug action (e.g., pharmacologic, PD) and effect in humans (including off-target)
29. Recognizes the pharmacological, biological, and statistical aspects of “fit-for purpose” biomarkers in drug development and regulatory evaluation
Domain: Quantitative Methods
30. Applies knowledge of E/R analyses to support development and review decisions
31. Recognizes limitations/potential pitfalls of various M&S approaches; includes ability to assess probability of acceptance of a given approach by members of the review team
32. Demonstrates the ability to detect patterns and correlations and identification of sub-populations, etc.
Domain: Drug Safety
33. Applies knowledge of preclinical toxicology and safety pharmacology (in vitro and in vivo) in human dose selection
34. Recognizes the safety margin and therapeutic index relative to the starting human dose/exposure, the target efficacious human dose/exposure and the potential effects at the expected human doses/exposures above the NOAEL or MABEL
35. Assesses safety in clinical studies; interprets results and their relevance for the risk–benefit balance
Domain: Pharmacotherapy
36. Exhibits knowledge of disease definition, etiology, pathophysiology, genetic variants (if any), and the spectrum of clinical phenotypes
37. Recognizes main treatment options/guidelines, indications, contra-indications, mechanism of action, side effects, limitations
38. Identifies the impact of disease variability on drug action and of drug variability on disease
Domain: Clinical Trial Methods
39. Applies the specific considerations for drug dosing, adverse effects, and measurement of efficacy in specific patient populations (e.g., neonates, infants, children, adolescents, elderly, renally/hepatically impaired, pregnant women)
Domain: Guidance and Policy
40. Demonstrates knowledge of the regulations and policies (e.g., MaPPs, IQPs) that apply to the review process (e.g., NDA/BLA, IND, Citizen Petitions)
41. Demonstrates knowledge and awareness of ICH and GCP requirements and applies where relevant
42. Applies Agency (U.S. and non-U.S.) guidances- relevant to clinical pharmacology and therapeutic product development
43. Identifies opportunities for new policy development to advance science-based, pragmatic drug development

The core competency domains are as follows: communication, time management, negotiation skills, problem solving, customer focus, and functional/skills. The core competencies consist of knowledge, skills, and abilities that all clinical pharmacologists in OCP should employ irrespective of their specialization. These competencies describe knowledge and interpersonal skills that staff need when collaborating with multi-disciplinary teams. The technical competency domains are as follows: drug disposition, pharmacology and biomarkers, quantitative methods, drug safety, pharmacotherapy, clinical trial methods, and guidance and policy. The technical competencies consist of foundational knowledge of pharmacology as well as specific subject matter and specialized skill sets.

Delphi studies are also successful when participants have a vested interest and are adequately prepared [8]. The number of respondents who participated, twenty-nine in total, was acceptable for a Delphi study [14]. The researcher prepared study participants by explaining the purpose of the study, the amount of time required, the number of study rounds, and the outcome of information gained from the study and those efforts yielded good results. The information gained from this study can be applied to education efforts within the office and contribute to strategic planning. Participants who volunteered were able to provide their input regarding competencies needed for their work success in the office. Furthermore, competency development directly aligns with one of the areas of the New Drugs Regulatory Program Modernization for the Agency and this work supports that initiative [18]. 

The diversity of the participant backgrounds also strengthened the study [17]. There was representation among all the divisions in OCP as seen in Tables 1 and 2. Participants had work history in different settings. Many participants had a greater number of years of experience in drug development than in OCP and the Agency. In round one 59.5% of participants had 11 or more years of experience in drug development. In comparison 46.9% of participants in round one had 0–5 years of experience in OCP and 43.8% of participants had 0–5 years of experience at the Agency. In round two 55.1% of participants had 11 or more years of experience in drug development. In comparison 41.4% of participants only had 0–5 years of experience at the Agency and 44.8% of participants had 0–5 years of experience in OCP. This suggests that participants may have had experience in the pharmaceutical industry settings or academia and that experience enabled them to rank the competencies. There were several participants who had many years of experience in the government setting. In round one 28.1% of participants had 11 plus years of experience in OCP. In round two 37.9% of participants had 11 or more years of experience in OCP. This mixture of participant experience added value to the study. Participants who had experience in drug development as both as a regulator and in industry had a unique perspective regarding competencies needed for success in the work setting.

## Limitations

Delphi studies by design inherently use purposive sampling and not randomized because they rely on the expertise of its participants. The study participants were from one office located in one agency. Although the number of the participants was within the accepted number for the Delphi technique [14], increasing the number to include participants from the entire office or clinical pharmacologists from other offices could further diversify the findings. The participants being from one office also posed challenges with inferential statistics. A McNemar test was conducted to show stability between rounds as referenced by von der Gracht [14]. A McNemar test was performed although the selection of participants was not a true random sample. The results demonstrated no significant difference between the two rounds thus showing stability in responses. Furthermore, the newly gained competencies included in round two were only ranked once because the study concluded in two rounds. Therefore, the McNemar test could not be performed and there was no comparison of the newly gained competencies. As consensus of 60% was used to determine the essential competencies, descriptive statistics were utilized for the results and conclusion of study. It should be noted that these findings are applicable for this particular office. [19] However, these study findings can influence the use of competencies in the workforce beyond OCP.

## Study Contributions/Implications for Practice

Our findings suggest core and technical competencies in the Office of Clinical Pharmacology can build essential training programs. This supports Brouwer et al. [2] who indicated training programs should include both specialized and essential competencies in clinical pharmacology and the importance of competency-based education [20]. These programs can enhance existing programs and provide an education framework to focus on staff development, scientific and regulatory training as well as pipeline development. Additionally, the Delphi method is a good consensus tool to determine essential competencies in clinical pharmacology in the regulatory environment. Regardless of technical background or specialization, the participants agreed on what they considered essential competencies for success in the workplace. An approach would be to utilize this information to enhance the knowledge, skills and, abilities of all OCP staff from beginner to experienced clinical pharmacologists. Since all core and technical competencies were deemed essential, the office could initially develop programs focusing on the core competencies, guidance competencies and the technical competencies receiving 80% or higher consensus until all of the competencies have been addressed [13]. These competencies can be a framework to establish and enhance education programs addressing workforce needs. Furthermore, competencies can be used to develop job profiles which specify the knowledge, skill, and abilities of suitable applicants [21]. 

Competencies are used for academic, self- assessment or professional purposes [21]. To further contribute to pipeline development universities could consider developing programs or curriculum that focus on the core and technical competencies in clinical pharmacology or other areas in drug development to develop the future workforce [20]. This can prepare students not only for academia, but other work settings such as industry or the government workforce [22]. Continued research is needed to align curriculum development to meet market demands promoting advancements in science [22]. Organizations could have a team dedicated to enhancing workforce development and education to remain abreast on ongoing research in competency development in pharmaceutical medicine and the drug development workforce to create competency-based programs.

It is important to establish a deliberate approach for developing scientists spanning from students to workforce professionals [5]. This study enhanced the previous knowledge matrix of core and technical clinical pharmacology competencies. There should be ongoing discussion about the use of competencies and relatedness to the emerging field because of the constant changes in drug development [21]. Competency development uses an iterative process and competencies need to be updated regularly since they have a span of 3–5 years to ensure the workforce has the necessary knowledge, skills, and abilities for optimal job performance [21]. Doing so prepares the workforce to handle the emerging landscape of drug development thus advancing public health.

## Conclusion

Essential core and technical competencies can build education programs to sustain the emerging clinical pharmacology workforce in the Office of Clinical Pharmacology. A well-planned Delphi study is a suitable consensus tool to determine essential clinical pharmacology competencies. Competencies can motivate standardization of skills and knowledge needed in clinical pharmacology to maintain a robust workforce.

### Supplementary Information

Below is the link to the electronic supplementary material.

Supplementary file1 (DOCX 22 kb)

Supplementary file2 (DOCX 21 kb)

## Data Availability

Not applicable.
