# Supplementary material for: A Modified Delphi Study to Establish Essential Clinical Pharmacology Competencies
Source: Ther Innov Regul Sci. 2024 Feb 6;58(3):473–82. doi: 10.1007/s43441-023-00609-y (PMC11043191; doi:10.1007/s43441-023-00609-y)
Supplement: Supplementary file 1 — Supplementary file1 (DOCX 22 kb) [file 43441_2023_609_MOESM1_ESM.docx]

**Appendix B (Online Resource 2)**

**Round Two Competencies**

***Round 2 Ranked Clinical Pharmacology Competencies***

|  |
| --- |

Core Competencies % Consensus

|  |
| --- |

Domain: Communication

1. Demonstrates effective writing and oral presentation/communication skills 100%
2. Appropriately adjusts communication material to optimize understanding and

utility by specific audiences (e.g., Industry, healthcare practitioner, public) 100%

Domain: Time Management

1. Uses time effectively and efficiently 96.6%
2. Prioritizes a wide range of tasks and projects in a timely manner 96.6%
3. Demonstrates the ability to plan the workload for successful completion of

projects 100%

Domain: Negotiation Skills

1. Demonstrates the ability to negotiate skillfully in tough situations with both internal

and external groups 93.1%

1. Seeks win-win solutions to settle differences 89.9%
2. Demonstrates skill in being direct and forceful as well as diplomatic

when required 96.6%

1. Builds trust between parties in negotiation 89.7%
2. Uses listening skills when negotiating* 96.4%

Domain: Problem Solving

1. Applies critical thinking to solve difficult problems with effective solutions 100%
2. Researches broadly to solve a problem and with an open mind 100%
3. Demonstrates sensitivity to potential unseen risks/threats during problem solving 96.6%
4. Provides an objective analysis 100%

Domain: Customer Service (Focus)

1. Applies knowledge of direct internal and external feedback for improvement in the drug development and regulatory review process, policy development and this information if communicated 96.6%
2. Strives to meet the needs, expectations and requirements of internal coworkers and external stakeholders (e.g., industry, healthcare providers, press, congress, public ) in a positive manner 89.6%
3. Establishes and maintains effective relationships with coworkers and stakeholders and gains their trust and respect 100%
4. Demonstrates dedication to stakeholder satisfaction 92.9%

Domain: Functional/Skills

1. Performs in depth Clinical Pharmacology and sub-specialty reviews, e.g., quantitative pharmacology (pharmacokinetics and genomics) submissions in IND, NDA, BLAs, Supplements and Amendments 96.6%
2. Provides independent recommendations to review team regarding information related to the approvability of a clinical pharmacology package 96.6%
3. Remains abreast/current in Clinical Pharmacology and new innovations in Clinical Pharmacology related to drug development 96.6%
4. Maintains basic knowledge of other disciplines as appropriate 93.1%
5. Applies up-to-date knowledge of Federal laws, FDA regulations, and related guidelines for industry applicable to the review process 100%
6. Maintains relevant knowledge of CDER guidances, MaPPs and review division processes and procedures and OCP best practice documents 100%

Technical Competencies

Domain: Drug Disposition

1. Assesses the clinical relevance of drug substance and formulation attributes to guide approvability, dosing/administration 86.2%
2. Demonstrates knowledge of enzyme(s)/transporter(s) involved in drug disposition, including sources of variability 96.6%
3. Applies knowledge of preclinical ADME properties to guide early clinical development (INDs) or aid in regulatory interpretation of NDAs/BLAs 79.31%
4. Translates knowledge of vitro, in vivo, and in silico methods to predict human metabolism to guide decision-making (labeling, post-marketing requests, etc.) 89.3%
5. Evaluates and interprets effects of intrinsic and extrinsic factors on clinical PK 100%
6. Recognize that drug-drug interactions being translated to labeling through the understanding of the drug development program in certain therapeutic areas is essential to public health mission* 93.1%

Domain: Pharmacology and Biomarkers

1. Demonstrates knowledge of various measures (and methods) for assessment of drug action (e.g., pharmacologic, PD) and effect in humans (including off-target) 93.1%
2. Identifies drug- and disease-related biomarkers: molecular, cellular, genetic, histological, functional disease markers, and the spectrum of their application: diagnostic, prognostic, predictive, marker for response/toxicity 79.3%
3. Applies best-practices in the evaluation of biomarkers in all phases of drug development and evaluation (e.g., discovery, optimization, implementation) 79.3%
4. Recognizes the pharmacological, biological, and statistical aspects of “fit-for purpose” biomarkers in drug development and regulatory evaluation 82.8%
5. Identifies when to consider targeted biomarker variability, pediatric development variability or diversity of (ethnic/racial) population frequency of biomarker during negotiation of final communication in labeling with stakeholders* 79.3%

Domain: Quantitative Methods

1. Applies knowledge of E/R analyses to support development and review decisions 93.1%
2. Recognizes limitations/potential pitfalls of various M&S approaches; includes ability to assess probability of acceptance of a given approach by members

of the review team 89.7%

1. Identifies, critically evaluates, and concisely describes methodological issues in pharmacometric, PBPK, and other analyses to facilitate quantitative analysis by Modeling and Simulation where appropriate 79.3%
2. Demonstrates the ability to detect patterns and correlations and identification of sub-populations, etc. 93.1%
3. Demonstrates working knowledge of various individual and population-based

data analysis 82.8%

1. Applies knowledge of disease frequency and prevalence to understand the full spectrum of safety/efficacy evaluations for decision making and communication* 79.3%

Domain: Drug Safety

1. Applies knowledge of preclinical toxicology and safety pharmacology (in vitro and in vivo) in human dose selection 86.2%
2. Interprets relevance of results of preclinical toxicology and safety pharmacology (in vivo and in vitro) for use of an NME in humans 75.9%
3. Recognizes the safety margin and therapeutic index relative to the starting human dose/exposure, the target efficacious human dose/exposure and the potential effects at the expected human doses/exposures above the NOAEL or MABEL 82.8%
4. Differentiates between on-target and off-target effects; interprets likely mechanism of toxicity, its causality, and predictivity for occurrence in humans 69%
5. Assesses safety in clinical studies; interprets results and their relevance for the risk-benefit balance 82.1%
6. Utilizes the most appropriate metrics and outcomes in analyses by working closely with drug safety and clinical teams* 89.7%
7. Apply relevant drug safety competences in labeling* 75.9%

Domain: Pharmacotherapy

1. Exhibits knowledge of disease definition, etiology, pathophysiology, genetic variants (if any) and the spectrum of clinical phenotypes 85.2%
2. Shows awareness of key parameters for diagnosis and monitoring of temporal changes (e.g., disease progression) 77.8%
3. Identifies main tools and techniques used for measurement of disease status, classification, sub-classification 81.5%
4. Demonstrates familiarity with markers of disease activity or response to pharmacological intervention used 88.9%
5. Recognizes main treatment options/guidelines, indications, contra-indications, mechanism of action, side effects, limitations 81.48%
6. Recognizes primary research areas for new treatment modalities, and justification (newly discovered disease pathways, new technologies, etc.) 70.4%
7. Identifies the impact of disease variability on drug action and of drug variability on disease 85.2%

Domain: Clinical Trial Methods

1. Applies the specific considerations for drug dosing, adverse effects, and measurement of efficacy in specific patient populations (e.g., neonates, infants, children, adolescents, elderly, renally/hepatically impaired, pregnant women) 88.9%
2. Utilizes statistical/data analysis concepts related to the design and interpretation of clinical pharmacology and other studies 77.8%

Identifies all possible design options (trial designs, endpoint selection, patient selection, analysis approaches) 70.4%

Domain: Guidance and Policy

1. Demonstrates knowledge of the regulations and policies (e.g., MaPPs, IQPs) that apply to the review process (e.g., NDA/BLA, IND, Citizen Petitions) 92.9%
2. Demonstrates knowledge and awareness of ICH and GCP requirements and applies where relevant 82.2%
3. Applies Agency (U.S. and non-U.S.) guidances- relevant to clinical pharmacology and therapeutic product development 92.9%
4. Identifies opportunities for new policy development to advance science-based, pragmatic drug development 96.4%

*Note.* * new competency gained from Round 1
